# Reductions in hospital care among clinically vulnerable children aged 0–4 years during the COVID-19 pandemic

**DOI:** 10.1136/archdischild-2021-323681

**Published:** 2022-06-21

**Authors:** David Etoori, Katie L Harron, Louise Mc Grath-Lone, Maximiliane L Verfürden, Ruth Gilbert, Ruth Blackburn

**Affiliations:** 1 Institute of Health Informatics, University College London, London, UK; 2 Department of Population, Policy and Practice, Institute of Child Health, University College London, London, London, UK

**Keywords:** Covid-19, Child Health, Healthcare Disparities, Health services research

## Abstract

**Objective:**

To quantify reductions in hospital care for clinically vulnerable children during the COVID-19 pandemic.

**Design:**

Birth cohort.

**Setting:**

National Health Service hospitals in England.

**Study population:**

All children aged <5 years with a birth recorded in hospital administrative data (January 2010–March 2021).

**Main exposure:**

Clinical vulnerability defined by a chronic health condition, preterm birth (<37 weeks’ gestation) or low birth weight (<2500 g).

**Main outcomes:**

Reductions in care defined by predicted hospital contact rates for 2020, estimated from 2015 to 2019, minus observed rates per 1000 child years during the first year of the pandemic (March 2020–2021).

**Results:**

Of 3 813 465 children, 17.7% (one in six) were clinically vulnerable (9.5% born preterm or low birth weight, 10.3% had a chronic condition). Reductions in hospital care during the pandemic were much higher for clinically vulnerable children than peers: respectively, outpatient attendances (314 vs 73 per 1000 child years), planned admissions (55 vs 10) and unplanned admissions (105 vs 79). Clinically vulnerable children accounted for 50.1% of the reduction in outpatient attendances, 55.0% in planned admissions and 32.8% in unplanned hospital admissions. During the pandemic, weekly rates of planned care returned to prepandemic levels for infants with chronic conditions but not older children. Reductions in care differed by ethnic group and level of deprivation. Virtual outpatient attendances increased from 3.2% to 24.8% during the pandemic.

**Conclusion:**

One in six clinically vulnerable children accounted for one-third to one half of the reduction in hospital care during the pandemic.

## Abstract (precis)

While children with chronic conditions, prematurity or were born with low birthweight make up a sixth of children aged 0-4 years in England, they account of up to half of COVID-21 pandemic-related reductions in hospital contact (from Jan 2020 to March 2021).

**What is already known on this topic**

- Young children with chronic health conditions have high rates of hospital care.
- Rates of planned and unplanned hospital admissions and outpatient care rose steadily in the decade before the pandemic.
- Hospital care reduced sharply after the onset of the COVID-19 pandemic for all children.

**What this study adds**

- One in six clinically vulnerable children 0–4 years old accounted for one-third to one-half of reductions in hospital care during the pandemic in this age group.
- There were small differences in reductions between black and Asian ethnic groups (vs white) and for children in the most deprived quintile (vs least deprived).
- Planned care weekly rates (outpatient or admissions) for children with chronic conditions reduced sharply during the pandemic and returned to prepandemic levels only among infants.

**How this study might affect research, practice or policy**

- Research is needed to understand the reductions in planned care, the types of care or procedures affected and the short-term and long-term implications for children.
- Research is needed to ascertain whether contact rates have returned to previous levels since the ending of pandemic restrictions.
- Children in vulnerable groups will likely need targeted catch-up funding and resources to mitigate or prevent adverse outcomes accruing from the reductions we report.

## Introduction

Rates of hospital contact (outpatient attendances, planned and unplanned hospital admissions) among 0–4 year-olds are highest for infants and have increased steadily in England over the past decade.^1–3^ Hospital utilisation patterns differ markedly by age and clinical vulnerability: children born preterm, with low birth weight or a congenital anomaly, have substantially more admissions than other children.^4 5^

Hospital contacts reduced substantially during the COVID-19 lockdown,^6–9^ which likely most impacted high intensity users of planned hospital care, such as children born too early or too small or with underlying health conditions.^1 2 10^ Postponed or cancelled planned hospital care may result in delayed diagnoses or treatments, which could be detrimental to health or development.^11^ Fewer unplanned hospital admissions might reflect fewer infections, injuries or other health problems due to reduced exposure during pandemic restrictions but could also reflect unmet need.

This study aimed to quantify reductions in planned and unplanned hospital care for clinically vulnerable children and non-vulnerable peers during the COVID-19 pandemic using national, longitudinal administrative hospital data for England. We measured planned hospital contacts (admissions and outpatient attendances) and unplanned admissions among children with chronic health conditions or born preterm or low birth weight and children with no recorded clinical vulnerability as well as by ethnicity and area-based deprivation. Reductions were quantified as the difference between predicted and observed rates of hospital contacts during the first year of the pandemic. We also examined whether rates of contact returned to prepandemic levels and described changes in the type of outpatient contact (eg, face to face or virtual).

## Methods

### Study population and data source

Children were included in the cohort if aged 0–4 years and their birth was recorded in hospital administrative data in the English NHS (Hospital Episode Statistics (HES)) between 1 January 2010 and 31 March 2021. HES records 97% of all births in England.^12^ Children were followed until the earliest of fifth birthday or 31 March 2021 (online supplemental figure 1). All contacts with NHS hospitals in England (including admitted patient care and outpatient attendances) were linked using the encrypted HES Identifier (ID). Accident and emergency (A&E) attendances could not be included as patient-level linkage is via Token ID.^13^ We combined consecutive consultant episodes and hospital transfers to form admissions.^14^

10.1136/archdischild-2021-323681.supp1 Supplementary data

### Outcome and exposure

Our primary outcome was the reduction in hospital care, defined as the difference between observed and predicted rates (per 1000 child-years (cy)) of hospital contacts (stratified into outpatient attendances, planned or unplanned admissions) during the pandemic (23 March 2020–22 March 2021). Inpatient admissions were classified as planned or unplanned using the ‘admission method’ variable (admissions have a predictable clinical need where the decision to admit may be separated in time from the actual admission).^15^ We also described trends in weekly rates of hospital contacts by age (from 1 January 2020 to 31 March 2021 and averaged for 2015–2019), and uptake (attended, missed, cancelled and postponed) and mode (in person, virtually) of outpatient contacts.

Clinical vulnerability was defined by a chronic condition recorded up to age 4 years, or preterm birth (<37 weeks of gestation) or low birth weight (<2500 g) recorded in birth or delivery records. A child had a chronic condition at a given age if at least one relevant International Classification of Diseases 10th revision (ICD-10) code (identified using code lists developed by Feudtner and Hardelid^12 16–18^) was recorded in any of their records up to that age including their birth admission. Completeness of demographic variables declined for children born during the pandemic (missingness increase by; 9% Index of Multiple Deprivation (IMD); 3% birth weight; 2% gestational age) but was unchanged for admission characteristics including inpatient admission type (planned, unplanned, birth or maternity) or outpatient attendance type (in-person and virtual), similar to outpatient data metrics published by NHS digital.^19^ Children with a chronic condition but missing gestation and birth weight were categorised as clinically vulnerable (chronic condition only).

We analysed risk factors associated with health service use: age (0–11 months, 1–4 years),^3^ quintile/fifth of deprivation derived from the national distribution of the IMD 2004 (an area measure for ~650 households)^20 21^ and recorded ethnic group (grouped as white, black, Asian or other, including mixed and Chinese).

### Statistical analyses

We calculated observed rates of hospital contacts per 1000 cy in the prepandemic period (2015–2019), stratifying by risk factors.

We calculated child-years at risk by averaging the eligible population of births recorded in HES at the beginning and end of a year, assuming no emigration and ignoring deaths. We used Poisson regression (including a linear effect of time and log of the midyear population as an offset) to model rates stratified by risk factors from 1 January 2015 to 31 December 2019. Data from 1 January to 22 March 2020 were excluded from the prepandemic period because reductions in hospital contact rates preceded the first lockdown. To calculate the reduction in hospital contacts, we predicted rates for the pandemic period, assuming that the pandemic had not occurred and previous trends would have continued. The reduction was estimated as the difference between predicted and observed rates. We also calculated reductions within the first national lockdown (23 March–23 June 2020), easing of restrictions (24 June–4 November 2020), second national lockdown (5 November–31 December 2020) and third national lockdown (1 January–22 March 2021).^22^

We calculated weekly rates between 1 January 2015 and 31 March 2021, using dynamic denominator study populations to accommodate temporal changes in hospital activity. Analyses stratified children with and without chronic conditions (recorded between birth and the relevant week) because we expected the differences to be largest between these comparators. Weekly rates of hospital contact were calculated by dividing the total number of weekly admissions or attendances by the weekly dynamic denominator population of children within each stratification level (ie, a child born in week 1 of 2015 would move into the 1-year-old group in week 1 of 2016 and age out of the cohort in week 1 of 2019). Weekly rates in 2020 and 2021 were plotted against average weekly rates for 2015–2019. We also modelled weekly rates between 1 January 2015 and 31 December 2019, using a Poisson model that included a linear effect of time, calendar month to account for seasonality, log of the weekly denominator population as an offset and second-order lagged residuals. A similar approach was used to estimate weekly reductions during the pandemic. Analyses were performed in Stata V.16.^23^

## Results

### Population characteristics

Of the 3 813 465 children aged 0–4 years, 394 384 (10.3%) had a record indicating a chronic condition (including congenital anomalies); 363 950 (9.5%) were born preterm or low birth weight; and 83 283 (2.2%) had both vulnerabilities. Overall, 675 051 (17.7%) had one or more of these clinical vulnerabilities (table 1).

**Table 1:** Demographic characteristics of children born between 1 January 2015 and 31 March 2021 by vulnerability status

	None (n=2 773 420; 72.7%)		LBW/preterm only (n=280 667; 7.4%)		CC only (n=311 101; 8.25)		Both (n=83 283; 2.2%)		Missing (n=364 994; 9.6%)		Total (n=3 813 465)	
	n	%	n	%	n	%	n	%	n	%	n	%
Sex												
Male	1 406 322	50.7	134 871	48.1	183 964	59.1	46 304	55.6	183 986	50.4	1 955 447	51.3
Female	1 366 276	49.3	145 481	51.8	127 006	40.8	36 860	44.3	180 806	49.5	1 856 429	48.7
Missing	822	0.03	315	0.1	131	0.04	119	0.1	202	0.1	1589	0.04
Age												
Infants	400 330	14.4	36 445	13.0	27 641	8.9	8317	10.0	80 502	22.1	553 235	14.5
1–4 years old	2 373 090	85.6	244 222	87.0	283 460	91.1	74 966	90.0	284 492	77.9	3 260 230	85.5
Ethnicity												
White	1 958 159	70.6	184 525	65.8	228 023	73.3	58 366	70.1	227 775	62.4	2 656 848	69.7
Black/black British	126 067	4.5	15 477	5.5	14 150	4.6	4681	5.6	14 924	4.1	175 299	4.6
Asian/Asian British	295 850	10.7	41 516	14.8	33 610	10.8	10 857	13.0	36 159	9.9	417 992	11.0
Any other ethnic groups	239 079	8.6	24 085	8.6	25 135	8.1	6633	8.0	34 579	9.5	329 511	8.6
Missing	154 265	5.6	15 064	5.4	10 183	3.3	2746	3.3	51 557	14.1	233 815	6.1
IMD												
Q1 (most deprived)	668 113	24.1	83 204	29.7	89 425	28.7	27 254	32.7	71 436	19.6	939 432	24.6
Q2	550 124	19.8	60 337	21.5	67 853	21.8	18 629	22.4	62 541	17.1	759 484	19.9
Q3	468 995	16.9	45 794	16.3	55 924	18.0	14 117	17.0	55 868	15.3	640 698	16.8
Q4	413 514	14.9	37 446	13.3	47 670	15.3	11 287	13.6	47 604	13.0	557 521	14.6
Q5 (least deprived)	400 528	14.4	34 530	12.3	43 052	13.8	10 119	12.2	35 619	9.8	523 848	13.7
Missing	272 146	9.8	19 356	6.9	7177	2.3	1877	2.3	91 926	25.2	392 482	10.3
Birth year												
2015	457 046	16.5	46 590	16.6	68 814	22.1	16 021	19.2	55 578	15.2	644 049	16.9
2016	471 324	17.0	49 020	17.5	63 505	20.4	16 042	19.3	44 186	12.1	644 077	16.9
2017	465 975	16.8	49 358	17.6	55 396	17.8	15 096	18.1	45 863	12.6	631 688	16.6
2018	443 438	16.0	47 406	16.9	47 628	15.3	13 730	16.5	56 695	15.5	608 897	16.0
2019	431 854	15.6	41 713	14.9	40 107	12.9	11 646	14.0	65 828	18.0	591 148	15.5
2020	409 956	14.8	38 128	13.6	30 598	9.8	9341	11.2	77 409	21.2	565 432	14.8
2021	93 827	3.4	8452	3.0	5053	1.6	1407	1.7	19 435	5.3	128 174	3.4

CC, chronic conditions; IMD, Index of Multiple Deprivation; LBW, low birth weight.

### Hospital contacts prepandemic

Hospital contacts were much higher among infants than children aged 1–4 years: 60.1% (95% CI 60.0 to 60.3) of infants and 8.2% (8.1–8.2) of 1–4 year-olds had ≥1 outpatient attendance each year, reflecting 1538 outpatient attendances per 1000 cy for infants and 302 for 1–4 year-olds (online supplemental figure 2, online supplemental table 1). Overall, 31.2% (31.1–31.3) of clinically vulnerable children had ≥1 outpatient attendance compared with 14.1% (14.1–14.2) of those with no known vulnerability reflecting 1483 attendances per 1000 cy for children with any vulnerability and 295/1000 cy for those without. Patterns were similar for planned and unplanned hospital admissions. Children with chronic conditions had the highest rates of admissions across all strata (online supplemental figures 3 and 4). Children born preterm or low birth weight but with no chronic condition had similar admission rates to their peers born at term or weighing ≥2500 g (online supplemental figure 2).

### Hospital contacts during the pandemic

There were stark reductions in rates of all hospital contact types during the pandemic (table 2, online supplemental tables 2-4). Reductions were much larger for children with a chronic condition (outpatient: −492 (−505 to –480) contacts per 1000cy; planned: −91/1000 cy (−95 to –86); unplanned: −230/1000 cy (−236 to –224)) than those without and particularly high for children with a chronic condition who were also born preterm or with a low birth weight (outpatient: −536/1000 cy (−563 to –508); planned: −105/1000 cy (−113 to –97); unplanned: −279/1000 cy (−290 to –268)). Relative reductions were lower for children with any known vulnerability compared with those without any known vulnerability (outpatient: −19.0% (−19.0 to −18.9) vs −25.7% (−25.8 to −25.6); planned: −27.6% (−27.9 to −27.3) vs −58.0% (−58.5 to −57.6); unplanned: −45.9% (−46.1 to –45.7) vs −69.4% (−69.6 to −69.3)) (table 2). The 17.7% of children identified as clinically vulnerable accounted for 50.1% (49.9–50.3) of reductions in outpatient attendances, 55.0% (54.6–55.5) in planned hospital admissions and 32.8% (32.6–33.0) in unplanned hospital admissions (table 2).

**Table 2:** Difference between predicted and observed rates of hospital contact during the pandemic (March 2020–2021) among children aged 0–4 years by clinical vulnerability group

	Percentage of children seen			Number of hospital contacts			% change	Rates per 1000 child-years			% change
	n	N	%	Predicted	Observed	Difference		Predicted	Observed	Difference (95% CI)	
Outpatient attendances											
Total*	370 623	2 765 941	13.4	1 500 674	1 173 091	−327 583	−21.8	543	424	−118 (−115 to 122)	−21.8
No known vulnerability	232 976	2 243 088	10.4	635 226	471 743	−163 483	−25.7	283	210	−73 (−71 to 74)	−25.7
Any vulnerability	137 647	522 853	26.3	865 448	701 348	−164 100	−19.0	1655	1341	−314† (−302 to 326)	−19.0
LBW/preterm only	29 622	226 439	13.1	94 660	76 039	−18 621	−19.7	418	336	−82† (−77 to 88)	−19.7
CC only	86 825	231 671	37.5	586 026	475 226	−110 800	−18.9	2530	2051	−478† (−465 to 492)	−18.9
Both	21 200	64 743	32.7	184 762	150 083	−34 679	−18.8	2854	2318	−536† (−508 to 563)	−18.8
Any CC‡	107 762	296 414	36.4	771 219	625 309	−145 910	−18.9	2602	2110	−492† (−480 to 505)	−18.9
Planned admissions											
Total	52 280	2 765 941	1.9	144 980	92 618	−52 362	−36.1	52	33	−19 (−18 to 20)	−36.1
No known vulnerability	15 287	2 243 088	0.7	40 568	17 022	−23 546	−58.0	18	8	−10 (−10 to 11)	−58.0
Any vulnerability	36 993	522 853	7.1	104 412	75 596	−28 816	−27.6	200	145	−55† (−51 to 59)	−27.6
LBW/preterm only	1389	226 439	0.6	3675	1522	−2153	−58.6	16	7	−10 (−8 to 11)	−58.6
CC only	31 038	231 671	13.4	85 367	65 506	−19 861	−23.3	368	283	−86† (−81 to 91)	−23.3
Both	4566	64 743	7.1	15 370	8568	−6802	−44.3	237	132	−105† (−97 to 113)	−44.3
Any CC	35 603	296 414	12.0	100 929	74 074	−26 855	−26.6	341	250	−91† (−86 to 95)	−26.6
Unplanned admissions											
Total	131 134	2 765 941	4.7	442 174	179 366	−262 808	−59.4	160	65	−95 (−93 to 97)	−59.4
No known vulnerability	66 830	2 243 088	3.0	254 263	77 713	−176 550	−69.4	113	35	−79 (−78 to 80)	−69.4
Any vulnerability	64 304	522 853	12.3	187 911	101 653	−86 258	−45.9	359	194	−165† (−159 to 171)	−45.9
LBW/preterm only	6046	226 439	2.7	25 520	7228	−18 292	−71.7	113	32	−81 (−78 to 84)	−71.7
CC only	52 285	231 671	22.6	134 055	84 126	−49 929	−37.2	579	363	−216† (−209 to 222)	−37.2
Both	5973	64 743	9.2	28 336	10 299	−18 037	−63.7	438	159	−279† (−268 to 290)	−63.7
Any CC	58 248	296 414	19.7	162 648	94 425	−68 223	−41.9	549	319	−230† (−224 to 236)	−41.9

*268 256 (8.8%) children missing gestational age and birth weight data.

†Significantly different from children with no known vulnerability (5% level of significance).

‡Any CC combines CC only and both.

CC, chronic conditions; LBW, low birth weight.

**Figure 1:**
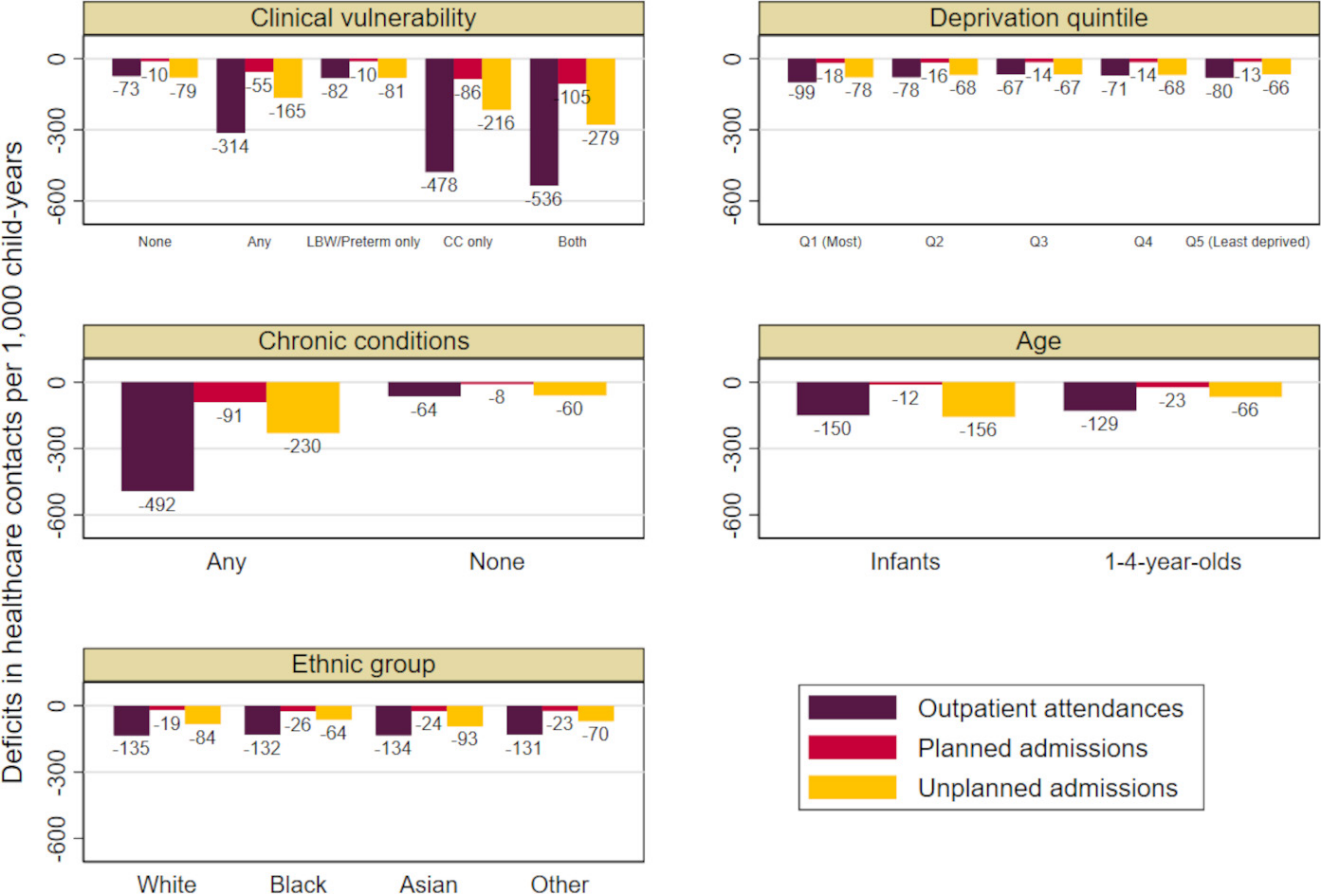
Reduction in care during the pandemic (March 2020–2021), estimated from predicted minus observed rates of hospital contacts per 1000 child-years for children aged 0–4 years, by clinical vulnerability status and risk factors. CC, chronic conditions; LBW, low birth weight.

Reductions were larger for infants than 1–4 year-olds for outpatient attendances and unplanned admissions but not for planned admissions (figure 1). We found small differences in reductions of planned and unplanned admissions across ethnic groups and in all hospital contacts among children in the most (vs least) deprived quintile (online supplemental tables 3 and 4). The largest reductions in care were among children with a chronic condition (online supplemental figures 5 and 6). Overall, the first lockdown was associated with the largest reductions in outpatient attendances and planned admissions. The largest reductions in unplanned admissions were seen in the second lockdown (table 3; online supplemental tables 5–7).

**Table 3:** Difference in predicted and observed rates of hospital contact per 1000 child-years among children aged 0–4 years during the pandemic (March 2020–2021) by period and clinical vulnerability

	First lockdown 23 March–23 June 2020		Easing of restrictions 24 June–4 November 2020		second lockdown Nov five to Dec 31, 2020		third lockdown Jan one to March 22, 2021	
	Reduction (95% CI)	% change	Reduction (95% CI)	% change	Reduction (95% CI)	% change	Reduction (95% CI)	% change
Outpatient attendances								
Total	−167 (−163 to 170)	−30.7	−88 (−86 to 91)	−16.3	−96 (−91 to 100)	−17.9	−128 (−123 to 132)	−23.5
No known vulnerability	−106 (−104 to 107)	−37.2	−55 (−54 to 56)	−19.5	−56 (−55 to 58)	−20.6	−75 (−73 to 77)	−26.2
Any vulnerability	−428* (−417 to 439)	−25.9	−230* (−221 to 240)	−13.9	−264* (−249 to 278)	−16.0	−352* (−337 to 367)	−21.4
LBW/preterm only	−121* (−116 to 127)	−28.7	−57 (−53 to 61)	−13.8	−73* (−67 to 80)	−18.0	−84 (−77 to 92)	−19.9
CC only	−664* (−651 to 677)	−26.2	−357* (−346 to 368)	−14.0	−398* (−381 to 414)	−15.9	−516* (−499 to 533)	−20.8
Both	−655* (−629 to 681)	−23.3	−385* (−364 to 407)	−13.7	−450* (−416 to 483)	−15.5	−699* (−665 to 735)	−24.2
Any CC	−663* (−652 to 675)	−25.5	−364* (−354 to 374)	−14.0	−410* (−395 to 425)	−15.8	−559* (−544 to 574)	−21.7
Planned admissions								
Total	−32 (−31 to 33)	−59.2	−16 (−15 to 17)	−29.3	−9 (−8 to 11)	−19.2	−16 (−15 to 17)	−31.9
No known vulnerability	−14 (−14 to 15)	−79.1	−9 (−9 to 9)	−48.5	−7 (−6 to 7)	−38.9	−11 (−11 to 12)	−62.0
Any vulnerability	−105* (−101 to 109)	−51.6	−46* (−43 to 49)	−22.0	−22* (−18 to 26)	−11.6	−36* (−32 to 41)	−19.4
LBW/preterm only	−12* (−11 to 13)	−75.3	−8 (−7 to 9)	−49.4	−6 (−5 to 7)	−42.1	−11 (−11 to 10)	−64.9
CC only	−185* (−180 to 190)	−49.0	−71* (−67 to 75)	−18.4	−24* (−18 to 30)	−6.9	−40* (−34 to 46)	−11.6
Both	−144* (−137 to 152)	−60.3	−86* (−80 to 93)	−35.8	−70* (−61 to 79)	−31.6	−114* (−105 to 125)	−48.2
Any CC	−177* (−172 to 181)	−50.8	−75* (−71 to 78)	−21.2	−35* (−30 to 40)	−10.7	−57* (−52 to 62)	−17.7
Unplanned admissions								
Total	−88 (−87 to 90)	−60.0	−77 (−76 to 79)	−51.9	−140 (−138 to 143)	−66.2	−99 (−97 to 101)	−64.2
No known vulnerability	−68 (−67 to 69)	−66.5	−66 (−66 to 67)	−63.2	−116 (−115 to 118)	−75.3	−84 (−83 to 85)	−76.7
Any vulnerability	−176* (−171 to 182)	−51.6	−124* (−120 to 128)	−36.9	−245* (−237 to 253)	−53.2	−161* (−154 to 168)	−47.1
LBW/preterm only	−69 (−66 to 72)	−68.0	−64 (−62 to 66)	−64.1	−125* (−121 to 130)	−78.3	−90* (−86 to 94)	−79.9
CC only	−263* (−257 to 270)	−47.2	−157* (−152 to 162)	−28.5	−309* (−300 to 318)	−43.1	−189* (−181 to 197)	−34.8
Both	−241* (−231 to 251)	−59.1	−216* (−208 to 225)	−54.8	−434* (−419 to 450)	−73.1	−311* (−298 to 325)	−72.8
Any CC	−259* (−254 to 265)	−49.3	−171* (−166 to 175)	−33.0	−337* (−330 to 345)	−48.8	−217* (−210 to 224)	−41.8

*Significantly different from children with no known vulnerability (5% level of significance).

CC, chronic conditions; LBW, low birth weight.

### Trends in hospital contacts

**Figure 2:**
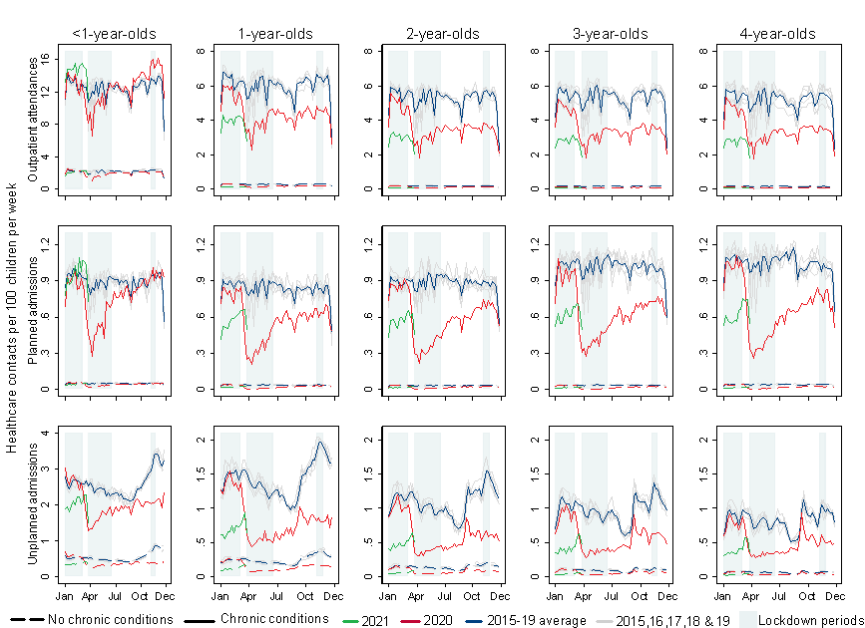
Weekly rates of hospital contacts among children aged 0–4 years during the pandemic (March 2020–2021) and averaged for 2015–2019 by age and presence of a chronic condition. Note: lockdown 1: 23 March–23 June 2020; lockdown 2: 5 November–6 December 2020; lockdown 3: 1 January–8 March 2021.

Outpatient attendances reduced sharply before and during the first national lockdown, among children of all ages with a chronic condition, with less perceptible changes among those without a chronic condition. Outpatient attendances rapidly returned to prepandemic rates for infants but remained below 2015–19 averages for older children. Planned admissions followed a similar pattern, with a return to prepandemic rates only for infants (figure 2).

A similar pattern was observed for reductions in rates of unplanned admissions, and these remained below prepandemic levels for both groups at all ages (figure 2). In 2020, the autumn–winter peak in unplanned admissions was diminished relative to previous years; however, following the reopening of primary schools at the end of the third lockdown on 8 March 2021, there was an increase in unplanned admission rates for all children, particularly those with a chronic condition (figure 2, online supplemental figure 7). Trends did not consistently differ across deprivation levels (online supplemental figures 8 and 9) or by ethnic group (online supplemental figures 10–12).

For all age groups, a spike in cancellations and postponement of outpatient appointments preceded the first lockdown by 3 weeks (online supplemental figure 13). There was an increase in tele/virtual outpatient attendances during the pandemic and face-to-face visits did not return to prepandemic levels in any age group (online supplemental table 8 and figure 14).

## Discussion

This population-based cohort study of all children aged <5 years in England found large and disproportionate reductions in planned and unplanned hospital contacts during the COVID-19 pandemic for clinically vulnerable groups. The one in six clinically vulnerable children accounted for over half the reduction in outpatient attendances and planned admissions, and one-third of the reduction in unplanned admissions. While absolute reductions were larger for vulnerable children, they were smaller in relative terms compared with children with no recorded vulnerability suggesting that hospitals prioritised these children. We saw some evidence of recovery in planned care during the pandemic among infants, but not among older children.

This study’s main strength is the use of a birth cohort of all children born in an NHS hospital in England (97% of all births). This large sample size gave us enough data to calculate weekly rates of hospital contacts. The longitudinal nature of the data allowed us to identify chronic conditions from diagnostic codes recorded in all admissions since birth, using a clinically developed coding system.^16^

Limitations include underascertainment of chronic conditions for children who could not be admitted to hospital due to the pandemic. These children may have been managed in primary care, or as outpatients, where chronic conditions coding is mostly missing. Furthermore, older children would have had more time for chronic conditions to be diagnosed. This likely explains the decline in prevalence of chronic conditions in more recent years. Analyses were restricted by variables available in HES (eg, use of IMD 2004). Vulnerability may be underestimated for the 10% of children who were excluded from the study due to missing gestational age and birth weight data. Multiple imputation of missing data was not feasible given the study size. We could not quantify the reduction in A&E attendances, as this dataset is not yet linkable to admission records. However, recent studies investigating A&E attendances in children reported similar reduced service use during the pandemic.^24 25^ Our rates did not account for deaths in the denominator (0.5%), non-NHS healthcare or emigration, but these events are rare. Our modelling approach required several assumptions, and our estimates of impacts are likely conservative.

Reductions in hospital care for children during the pandemic have been reported in Europe,^7 26–33^ Asia,^34^ North^35–38^ and South America.^39^ Most studies investigated A&E attendances or unplanned admissions.^25–39^ Other studies report a reduction in asthma-related paediatric emergency department attendances^29^ and reduced likelihood of admission, assessment and surgery for children with epilepsy.^40^ Furthermore, significant reductions in infection-related hospitalisations have been observed,^30 34 36 41^ particularly for children under 5 years.^41^ Two studies conducted national level analyses.^30 32^ We believe our study reports the first population-level reductions in planned care (admissions and outpatient) for children with health risk-factors for a full year of the pandemic. Previous research in adult populations has reported on the disproportionate burden of COVID-19 infection, hospitalisation and death in minority ethnic groups.^42 43^ Our study did not examine COVID-19 related contacts because hospitalisation is rare as children typically experience mild asymptomatic disease.^44 45^ However, we identified small differences in reductions of hospital care for children in the Asian ethnic group and for children in the most deprived quintile. This suggests that inequalities exacerbated by COVID-19 in adult populations might also extend to children from more deprived, minority ethnic backgrounds.

Potential mechanisms underpinning reductions in planned care likely represent restrictions to access, supported by a rise in postponed outpatient care. Our findings show these restrictions were mitigated in infants, who have a high frequency of hospital care and for whom interventions are likely to be more time critical than in older children.^3^ However, reductions remained large in older children and may reflect unmet need or postponed care that could have longer term health consequences.^11^ A move to virtual appointments may have exacerbated existing inequalities for families without access to the internet at home.

Reductions in unplanned care may be driven by opposing factors. Previous studies reported decreases in unplanned infection-related hospitalisations due to reduced social exposure and increased hygiene, with little change in admissions for non-infectious causes like appendicitis.^27 30 31 34 41^ Others have reported reductions in injury.^46^ The spike in unplanned admissions after schools reopened in autumn 2020 and in March 2021 when the third lockdown ended likely reflects increased socialisation. Other positive effects could include reduced exposure to triggers for respiratory disease (eg, air pollution)^47^ and improved medication adherence through increased parental supervision. Negative implications could include reduced extrinsic interventions through education, health and social care professionals,^7 48^ or delaying or avoiding medical care due to fears of hospital-acquired COVID-19 infection.^38 49–51^ Additionally, these reductions could represent missed opportunities for earlier and more effective intervention.^11 52^

This analysis was the first step in quantifying deferred or foregone hospital care during the pandemic. Studies using routine administrative data report only acute hospital presentations,^37 38^ which may reflect late or missed diagnoses.^11 52^ Our findings confirm and quantify the reduction in hospital contacts for preschool children in England during the first year of the COVID-19 pandemic. Research is needed to understand reductions in planned care, the types of care, procedures or treatments affected and the short-term and long-term implications for children with specific conditions. More research will be needed to ascertain whether contact rates have returned to previous levels since the end of restrictions. Further research will also be needed to identify vulnerable groups likely to experience adverse outcomes from unmet healthcare in order to target ‘catch-up’ funding and resources to prevent or mitigate these adverse outcomes.^53^

## Data Availability

Data may be obtained from a third party and are not publicly available. The data used in this analysis are expected to be available to accredited researchers in 2022 (as part of the ECHILD Database) by applying to the data providers (DfE and NHS Digital).
