# Supplementary material for: Reductions in hospital care among clinically vulnerable children aged 0–4 years during the COVID-19 pandemic
Source: Arch Dis Child. 2022 Jun 20:archdischild-2021-323681. doi: 10.1136/archdischild-2021-323681 (PMC9271837; doi:10.1136/archdischild-2021-323681)
Supplement: Supplementary file 1 [file archdischild-2021-323681supp001.pdf]

Supplementary Table 1: Average percentage of 0–4-year-olds with at least one hospital contact per year (2015-19), by risk factors.

	N (%)	Outpatient attendances	Planned admissions	Unplanned admissions
**Total**	3,230,549*			
**Clinical vulnerability**				
No known vulnerability	2,333,773 (72.2)	14.1 (14.1, 14.2)	1.7 (1.7, 1.7)	8.8 (8.7, 8.8)
Any vulnerability	644,933 (20.0)	31.2 (31.1, 31.3)	10.3 (10.2, 10.3)	18.4 (18.3, 18.5)
• LBW/Preterm only	238,559 (7.4)	17.8 (17.6, 17.9)	1.6 (1.6, 1.7)	8.4 (8.3, 8.5)
• CC only	329,072 (10.2)	39.1 (39.0, 39.3)	16.0 (15.9, 16.2)	25.3 (25.2, 25.5)
• Both	77,302 (2.4)	38.8 (38.4, 39.1)	12.3 (12.1, 12.5)	20.0 (19.7, 20.3)
Any CC[α]	406,374 (12.6)	38.9 (38.8, 39.1)	15.3 (15.2, 15.4)	24.3 (24.2, 24.4)
Missing	251,843 (7.8)	—	—	—
**Age**				
Infants (<1 year old)	628,900 (19.5)	60.1 (60.0, 60.3)	4.2 (4.1, 4.2)	26.4 (26.3, 26.5)
1–4-year-olds	2,601,649 (80.5)	8.2 (8.1, 8.2)	3.5 (3.4, 3.5)	8.0 (7.9, 8.0)
**Ethnic group**				
White	2,296,986 (71.1)	17.7 (17.7, 17.8)	3.7 (3.7, 3.7)	12.0 (12.0, 12.0)
Black	153,009 (4.7)	15.8 (15.6, 16.0)	3.8 (3.7, 3.9)	9.2 (9.0, 9.3)
Asian	345,843 (10.7)	18.0 (17.9, 18.2)	3.6 (3.5, 3.7)	11.6 (11.5, 11.7)
Other	257,651 (8.0)	19.1 (19.0, 19.3)	3.6 (3.5, 3.7)	10.7 (10.5, 10.8)
Missing	177,060 (5.5)	—	—	—
**Deprivation quintile**				
Q1 (most deprived)	755,351 (23.4)	19.5 (19.4, 19.6)	4.4 (4.3, 4.4)	14.0 (13.9, 14.1)
Q2	607,815 (18.8)	19.6 (19.5, 19.7)	4.2 (4.2, 4.3)	13.3 (13.2, 13.4)
Q3	502,672 (15.6)	20.2 (20.1, 20.3)	4.2 (4.1, 4.2)	13.5 (13.4, 13.6)
Q4	430,486 (13.3)	21.2 (21.1, 21.3)	4.3 (4.2, 4.3)	13.8 (13.6, 13.9)
Q5 (least deprived)	404,753 (12.5)	22.5 (22.3, 22.6)	4.3 (4.3, 4.4)	13.6 (13.5, 13.7)
Missing	529,472 (16.4)	—	—	—

LBW – Low birthweight; **CC** – Chronic conditions. * Average eligible population between 2015-2019. Ref – Reference group. P-values: Chi-square test for comparison with reference group. A Children with any chronic condition (combines CC only and Both).

Supplementary Table 2: Difference in predicted and observed rates of hospital contact during the pandemic (March 2020-2021) among children aged 0 to 4 years, by age.

	Percentage of children seen			Number of hospital contacts				Rates per 1000 child-years			
	n	N	%	Predicted	Observed	Difference	% change	Predicted	Observed	Difference (95% CI)	% change
**Outpatient attendances**											
**Total**	426,871	3,034,197	14.1	1,687,100	1,282,399	-404,701	-24.0	556	423	**-133** (-131, -136)	-24.0
Infants (<1 year old)	313,236	568,812	55.1	900993	815513	-85,480	-9.5	1584	1434	**-150*** (-143, -157)	-9.5
1–4-year-olds	113,635	2,465,385	4.6	786107	466886	-319,221	-40.6	319	189	**-129** (-128, -131)	-40.6
**Planned admissions**											
**Total**	63,439	3,034,197	2.1	166,954	104,464	-62,490	-37.4	55	34	**-21** (-20, -21)	-37.4
Infants (<1 year old)	19,597	568,812	3.4	35045	28108	-6,937	-19.8	62	49	**-12*** (-11, -14)	-19.8
1–4-year-olds	43,842	2,465,385	1.8	131909	76356	-55,553	-42.1	54	31	**-23** (-22, -23)	-42.1
**Unplanned admissions**											
**Total**	208,377	3,034,197	6.9	519,640	268,220	-251,420	-48.4	171	88	**-83** (-81, -84)	-48.4
Infants (<1 year old)	106,048	568,812	18.6	226396	137884	-88,512	-39.1	398	242	**-156*** (-152, -159)	-39.1
1–4-year-olds	102,329	2,465,385	4.2	293244	130336	-162,908	-55.6	119	53	**-66** (-65, -67)	-55.6

* Significantly different from 1–4-year-olds (5% level of significance).

Supplementary Table 3: Difference in predicted and observed rates of hospital contact during the pandemic (March 2020-2021) among children aged 0 to 4 years, by ethnic group.

	Percentage of children seen			Number of hospital contacts				Rates per 1000 child-years			
	n	N	%	Predicted	Observed	Difference	% change	Predicted	Observed	Difference (95% CI)	% change
**Outpatient attendances**											
**Total**$^{\alpha}$	380,925	2,872,476	13.3	1,589,161	1,203,043	-386,118	-24.3	553	419	**-134** (-131, -138)	-24.3
White	277,757	2,125,238	13.1	1177279	889991	-287,288	-24.4	554	419	**-135** (-133, -137)	-24.4
Black	17,582	140,621	12.5	70897	52346	-18,551	-26.2	504	372	**-132** (-124, -140)	-26.2
Asian	48,025	338,198	14.2	195379	150173	-45,206	-23.1	578	444	**-134** (-128, -139)	-23.1
Other	37,561	268,419	14.0	145606	110533	-35,073	-24.1	542	412	**-131** (-125, -137)	-24.1
**Planned admissions**											
**Total**	60,815	2,872,476	2.1	161,107	102,311	-58,796	-36.5	56	36	**-20** (-19, -22)	-36.5
White	45,439	2,125,238	2.1	116675	75721	-40,954	-35.1	55	36	**-19** (-19, -20)	-35.1
Black	3,053	140,621	2.2	8443	4798	-3,645	-43.2	60	34	**-26*** (-23, -29)	-43.2
Asian	6,790	338,198	2.0	20335	12319	-8,016	-39.4	60	36	**-24*** (-22, -26)	-39.4
Other	5,533	268,419	2.1	15654	9473	-6,181	-39.5	58	35	**-23*** (-21, -25)	-39.5
**Unplanned admissions**											
**Total**	199,183	2,872,476	6.9	498,620	260,250	-238,370	-47.8	174	91	**-83** (-81, -85)	-47.8
White	152,225	2,125,238	7.2	378452	199614	-178,838	-47.3	178	94	**-84** (-83, -85)	-47.3
Black	7,566	140,621	5.4	18489	9442	-9,047	-48.9	131	67	**-64*** (-60, -69)	-48.9
Asian	22,516	338,198	6.7	61164	29575	-31,589	-51.6	181	87	**-93*** (-90, -97)	-51.6
Other	16,876	268,419	6.3	40515	21619	-18,896	-46.6	151	81	**-70*** (-67, -74)	-46.6

* Significantly different from children in the white ethnic group (5% level of significance). Other ethnic group includes Chinese and mixed ethnicity. α 161,721 (5.3%) children missing ethnicity data.

Supplementary Table 4: Difference in predicted and observed rates of hospital contact during the pandemic (March 2020-2021) among children aged 0 to 4 years, by deprivation quintile.

	Percentage of children seen			Number of hospital contacts				Rates per 1000 child-years			
	n	N	%	Predicted	Observed	Difference	% change	Predicted	Observed	Difference (95% CI)	% change
**Outpatient attendances**											
**Total**[α]	389,098	2,733,692	14.2	1,433,837	1,213,380	-220,457	-15.4	525	444	**-81** (-78, -86)	-15.4
Q1	103,866	750,360	13.8	405740	331783	-73,957	-18.2	541	442	**-99*** (-95, -102)	-18.2
Q2	84,205	604,305	13.9	304926	257812	-47,114	-15.5	505	427	**-78** (-74, -82)	-15.5
Q3	72,458	512,075	14.1	260352	226099	-34,253	-13.2	508	442	**-67*** (-63, -71)	-13.2
Q4	65,403	447,272	14.6	234302	202578	-31,724	-13.5	524	453	**-71** (-67, -75)	-13.5
Q5 (least deprived)	63,166	419,680	15.1	228517	195108	-33,409	-14.6	545	465	**-80** (-75, -84)	-14.6
**Planned admissions**											
**Total**	62,744	2,733,692	2.3	146,454	104,255	-42,199	-28.8	54	38	**-16** (-14, -17)	-28.8
Q1	17,518	750,360	2.3	42046	28414	-13,632	-32.4	56	38	**-18*** (-17, -19)	-32.4
Q2	13,703	604,305	2.3	32566	22934	-9,632	-29.6	54	38	**-16*** (-15, -17)	-29.6
Q3	11,625	512,075	2.3	26400	19069	-7,331	-27.8	52	37	**-14** (-13, -16)	-27.8
Q4	10,163	447,272	2.3	23504	17253	-6,251	-26.6	53	39	**-14** (-13, -15)	-26.6
Q5 (least deprived)	9,735	419,680	2.3	21938	16585	-5,353	-24.4	52	40	**-13** (-11, -14)	-24.4
**Unplanned admissions**											
**Total**	206,549	2,733,692	7.6	459,218	267,418	-191,800	-41.8	168	98	**-70** (-68, -73)	-41.8
Q1	57,328	750,360	7.6	133406	74919	-58,487	-43.8	178	100	**-78*** (-76, -80)	-43.8
Q2	44,184	604,305	7.3	98003	57071	-40,932	-41.8	162	94	**-68** (-66, -70)	-41.8
Q3	38,374	512,075	7.5	84028	49672	-34,356	-40.9	164	97	**-67** (-65, -69)	-40.9
Q4	34,434	447,272	7.7	74938	44550	-30,388	-40.6	168	100	**-68** (-65, -71)	-40.6
Q5 (least deprived)	32,229	419,680	7.7	68843	41206	-27,637	-40.1	164	98	**-66** (-63, -68)	-40.1

* Significantly different from children in the least deprived quintile (5% level of significance). α 300,505 (9.9%) children missing IMD data

Supplementary Table 5: Difference in predicted and observed rates of hospital contact per 1,000 child-years among children aged 0 to 4 years during the pandemic (March 2020-2021), by period and age.

	March 23 to June 23, 2020		June 24 to Nov 4, 2020		Nov 5 to Dec 31, 2020		Jan 1 to March 22, 2021	
	Reduction (95% CI)	% change	Reduction (95% CI)	% change	Reduction (95% CI)	% change	Reduction (95% CI)	% change
**Outpatient attendances**								
**Total**	**-170** (-167, -172)	-30.7	**-90** (-88, -92)	-16.6	**-101** (-98, -104)	-18.8	**-184** (-181, -188)	-30.7
Infants (<1 year old)	**-310*** (-304, -317)	-20.2	**-37*** (-32, -43)	-2.4	**-70*** (-62, -79)	-4.5	**-207*** (-198, -217)	-12.3
1–4-year-olds	**-137** (-136, -139)	-43.9	**-102** (-101, -103)	-32.9	**-108** (-106, -110)	-36.0	**-179** (-177, -181)	-51.3
**Planned admissions**								
**Total**	**-33** (-32, -33)	-60.1	**-16** (-15, -17)	-29.1	**-9** (-8, -10)	-17.6	**-22** (-21, -23)	-38.2
Infants (<1 year old)	**-25*** (-23, -26)	-40.8	**-8*** (-7, -9)	-13.1	**-2*** (0, -4)	-3.3	**-12*** (-10, -14)	-18.3
1–4-year-olds	**-35** (-34, -35)	-65.2	**-18** (-17, -18)	-33.4	**-10** (-10, -11)	-21.6	**-24** (-24, -25)	-43.4
**Unplanned admissions**								
**Total**	**-82** (-81, -83)	-53.2	**-60** (-59, -61)	-38.2	**-124** (-122, -126)	-55.5	**-92** (-91, -94)	-52.1
Infants (<1 year old)	**-150*** (-147, -153)	-41.4	**-103*** (-100, -106)	-28.6	**-288*** (-283, -293)	-53.4	**-154*** (-149, -159)	-38.7
1–4-year-olds	**-66** (-65, -67)	-62.6	**-50** (-49, -50)	-45.4	**-86** (-85, -87)	-57.2	**-78** (-77, -80)	-61.8

* Significantly different from 1–4-year-olds (5% level of significance).

Supplementary Table 6: Difference in predicted and observed rates of hospital contact per 1,000 child-years among children aged 0 to 4 years during the pandemic (March 2020-2021), by period and ethnic group.

	March 23 to June 23, 2020		June 24 to Nov 4, 2020		Nov 5 to Dec 31, 2020		Jan 1 to March 22, 2021	
	Reduction (95% CI)	% change	Reduction (95% CI)	% change	Reduction (95% CI)	% change	Reduction (95% CI)	% change
**Outpatient attendances**								
**Total**	**-178** (-175, -181)	-34.1	**-99** (-97, -102)	-18.1	**-111** (-107, -115)	-20.5	**-157** (-153, -161)	-27.7
White	**-179** (-177, -181)	-32.3	**-99** (-97, -101)	-18.0	**-118** (-116, -121)	-21.7	**-155** (-153, -158)	-27.6
Black	**-178** (-170, -185)	-36.1	**-101** (-94, -107)	-20.1	**-67*** (-58, -76)	-14.1	**-175*** (-164, -186)	-32.5
Asian	**-179** (-174, -185)	-31.5	**-103** (-98, -107)	-17.9	**-87*** (-80, -93)	-15.8	**-163** (-156, -171)	-26.9
Other	**-176** (-170, -181)	-32.8	**-98** (-93, -102)	-18.1	**-105*** (-98, -112)	-19.9	**-150** (-142, -158)	-26.8
**Planned admissions**								
**Total**	**-34** (-33, -35)	-60.1	**-17** (-16, -18)	-29.4	**-9** (-8, -10)	-17.7	**-19** (-17, -20)	-33.5
White	**-33** (-32, -33)	-59.2	**-16** (-15, -16)	-27.8	**-9** (-8, -10)	-17.3	**-17** (-16, -18)	-31.4
Black	**-37*** (-34, -39)	-62.4	**-24*** (-22, -27)	-38.6	**-11** (-8, -15)	-20.7	**-26*** (-23, -30)	-44.0
Asian	**-38*** (-36, -39)	-61.7	**-20*** (-19, -22)	-33.5	**-10** (-8, -12)	-17.4	**-23*** (-21, -25)	-37.8
Other	**-39*** (-37, -41)	-63.8	**-18*** (-17, -20)	-30.7	**-10** (-8, -12)	-18.9	**-22*** (-19, -25)	-38.1
**Unplanned admissions**								
**Total**	**-85** (-83, -87)	-53.6	**-62** (-61, -63)	-38.4	**-128** (-125, -130)	-55.6	**-83** (-81, -85)	-48.9
White	**-84** (-83, -85)	-51.5	**-63** (-62, -64)	-38.2	**-135** (-134, -137)	-56.8	**-82** (-81, -84)	-47.6
Black	**-74*** (-70, -78)	-59.9	**-48*** (-45, -51)	-37.9	**-85*** (-80, -91)	-51.9	**-65*** (-60, -71)	-52.1
Asian	**-104*** (-101, -107)	-62.8	**-67*** (-65, -70)	-40.4	**-122*** (-118, -127)	-52.8	**-102*** (-98, -107)	-55.9
Other	**-77*** (-74, -80)	-55.5	**-54*** (-51, -56)	-37.7	**-95*** (-90, -99)	-49.5	**-72*** (-68, -76)	-48.7

* Significantly different from children in the white ethnic group (5% level of significance).

Supplementary Table 7: Difference in predicted and observed rates of hospital contact per 1,000 child-years among children aged 0 to 4 years during the pandemic (March 2020-2021), by period and deprivation quintile.

	March 23 to June 23, 2020		June 24 to Nov 4, 2020		Nov 5 to Dec 31, 2020		Jan 1 to March 22, 2021	
	Reduction (95% CI)	% change	Reduction (95% CI)	% change	Reduction (95% CI)	% change	Reduction (95% CI)	% change
**Outpatient attendances**								
**Total**	**-134** (-131, -138)	-25.4	**-51** (-48, -54)	-9.6	**-64** (-60, -69)	-12.4	**-80** (-75, -85)	-15.5
Q1	**-151*** (-148, -155)	-28.0	**-75*** (-72, -77)	-13.7	**-74*** (-70, -78)	-13.9	**-93** (-89, -98)	-17.6
Q2	**-126** (-123, -130)	-25.1	**-50** (-47, -53)	-9.9	**-64** (-60, -69)	-12.9	**-77*** (-72, -81)	-15.5
Q3	**-126** (-122, -129)	-24.4	**-34*** (-31, -37)	-6.7	**-49*** (-44, -54)	-9.7	**-65*** (-60, -70)	-13.1
Q4	**-127** (-123, -131)	-23.8	**-35*** (-32, -39)	-6.7	**-65** (-60, -70)	-12.5	**-69*** (-64, -74)	-13.6
Q5 (least deprived)	**-131** (-127, -136)	-23.9	**-45** (-42, -49)	-8.3	**-63** (-58, -69)	-11.9	**-87** (-82, -93)	-16.3
**Planned admissions**								
**Total**	**-31** (-30, -32)	-56.0	**-12** (-11, -13)	-21.8	**-5** (-3, -6)	-9.2	**-11** (-10, -13)	-21.8
Q1	**-33*** (-32, -35)	-58.2	**-14*** (-13, -15)	-24.8	**-7*** (-6, -9)	-14.0	**-15*** (-13, -16)	-26.9
Q2	**-31*** (-30, -32)	-56.5	**-13*** (-12, -14)	-22.8	**-4** (-2, -5)	-7.6	**-12*** (-11, -14)	-23.7
Q3	**-29** (-27, -30)	-55.1	**-12** (-11, -13)	-22.3	**-4** (-3, -6)	-8.6	**-8** (-7, -10)	-17.7
Q4	**-30** (-28, -31)	-55.1	**-10** (-9, -11)	-17.8	**-3** (-1, -4)	-5.6	**-11*** (-9, -12)	-21.7
Q5 (least deprived)	**-28** (-27, -29)	-53.0	**-10** (-9, -11)	-18.2	**-3** (-2, -5)	-6.7	**-6** (-5, -8)	-13.3
**Unplanned admissions**								
**Total**	**-76** (-74, -78)	-48.8	**-51** (-49, -53)	-32.3	**-116** (-113, -119)	-51.3	**-62** (-60, -65)	-39.9
Q1	**-88*** (-86, -90)	-52.7	**-56*** (-55, -58)	-33.9	**-122*** (-119, 125)	-51.9	**-70*** (-68, -73)	-42.0
Q2	**-77*** (-75, -79)	-50.2	**-48** (-46, -49)	-31.7	**-110** (-107, -113)	-50.3	**-60** (-57, -62)	-40.0
Q3	**-71** (-69, -73)	-47.2	**-47** (-45, -49)	-30.5	**-116** (-112, -119)	-51.9	**-61** (-58, -64)	-39.8
Q4	**-69** (-67, -71)	-45.2	**-51** (-49, -53)	-32.2	**-118** (-114, -121)	-51.8	**-59** (-56, -62)	-37.9
Q5 (least deprived)	**-67** (-65, -69)	-45.0	**-51** (-49, -53)	-32.4	**-110** (-107, -114)	-50.1	**-57** (-54, -60)	-37.9

* Significantly different from children in the least deprived quintile (5% level of significance).

Supplementary table 8: Type of outpatient attendance before and during the pandemic

	Pre-pandemic (Jan 1, 2015- Mar 22, 2020)		During the pandemic (Mar 23, 2020- Mar 31, 2021)	
Outpatient attendance type	n	%	n	%
Attended in-person	12,470,629	96.3	1,730,141	74.5
Attended tele/virtual	411,112	3.2	570,405	24.6
Missing	59,318	0.5	20,485	0.9
Total	12,941,059	100	2,321,031	100

Supplementary Figure 1: Study population showing ascertainment of vulnerability status (exposure) from birth and hospital contacts (outcomes) from January 1, 2015 to March 31, 2021 for children aged 0 to 4 completed years. *

						Outcome year						
Year of birth	2010	2011	2012	2013	2014	2015	2016	2017	2018	2019	2020	2021
2010	<1	1	2	3	4	5	6	7	8	9	10	11
2011		<1	1	2	3	4	5	6	7	8	9	10
2012			<1	1	2	3	4	5	6	7	8	9
2013				<1	1	2	3	4	5	6	7	8
2014					<1	1	2	3	4	5	6	7
2015						<1	1	2	3	4	5	6
2016							<1	1	2	3	4	5
2017								<1	1	2	3	4
2018									<1	1	2	3
2019										<1	1	2
2020											<1	1
2021												<1

Exposure

- Low birth weight and preterm births
- Chronic conditions
- 2 Age

Outcomes

- Not considered
- Partially considered
- Fully considered
- Not yet born

*Partially considered: For example, a child born on 1st June 2010 would turn 5 on 1st June 2015 and would therefore contribute data for part of 2015.

Supplementary Figure 2: Pre-pandemic average rate of hospital contacts per 1,000 child-years among children aged 0 to 4 years (2015 to 2019), by clinical vulnerability status and risk factors.

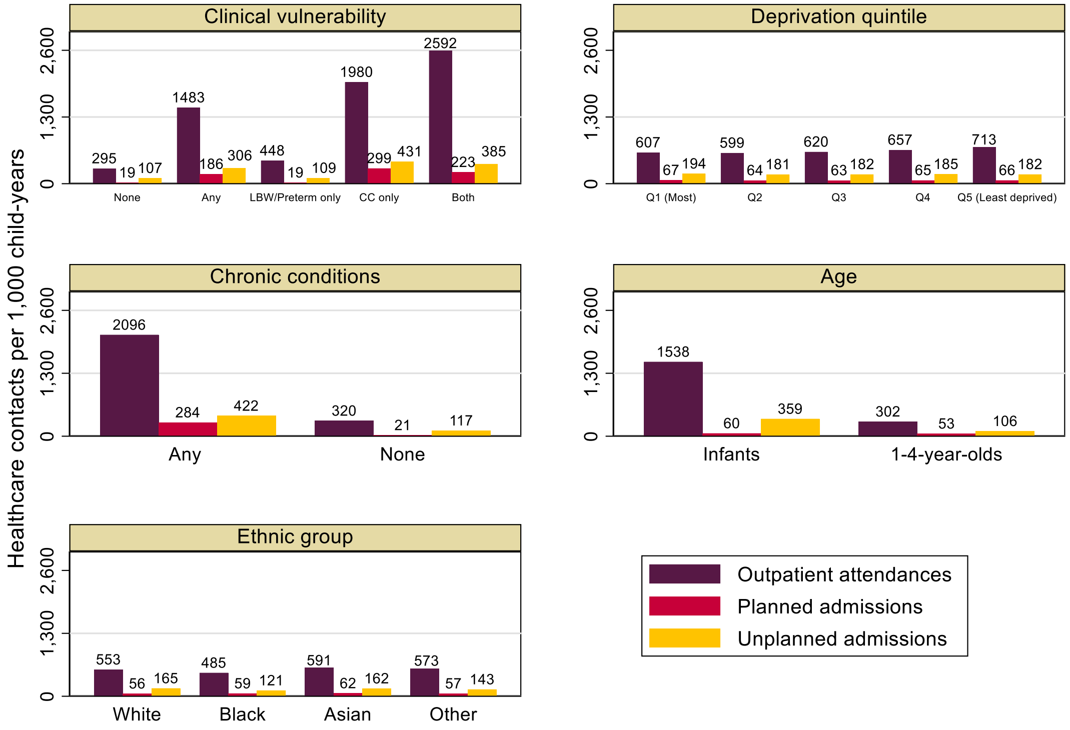

LBW – Low birthweight; **CC** – Chronic conditions

Supplementary Figure 3: Pre-pandemic average rate of hospital contacts per 1,000 child-years among children aged 0 to 4 years (2015 to 2019), by ethnic group and presence of a chronic condition.

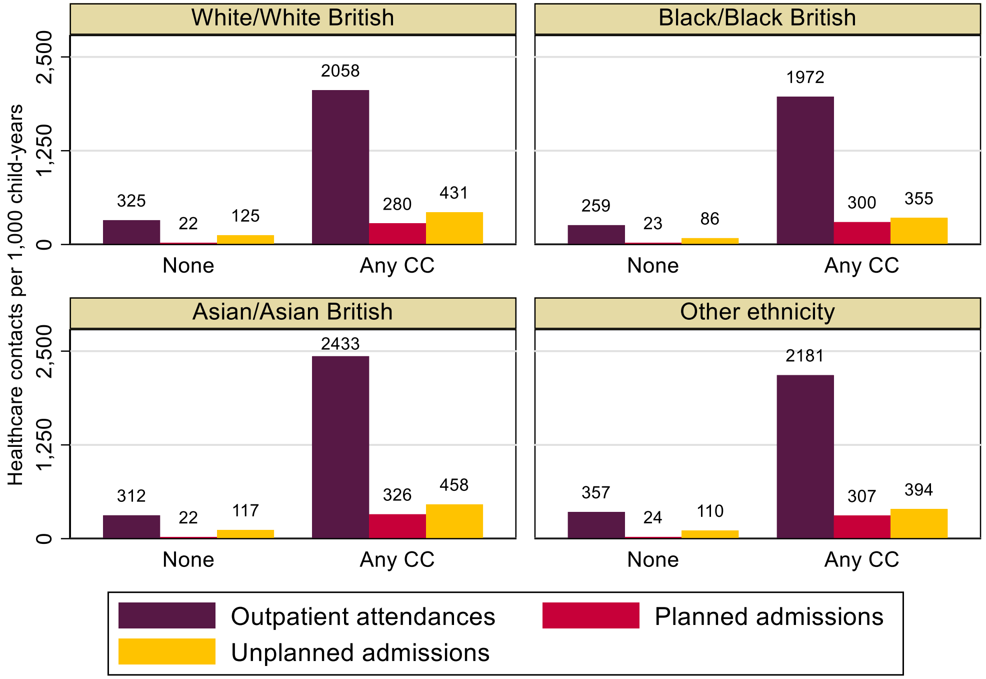

CC – Chronic conditions

Supplementary Figure 4: Pre-pandemic average rate of hospital contacts per 1,000 child-years among children aged 0 to 4 years (2015 to 2019), by deprivation quintile and presence of a chronic condition.

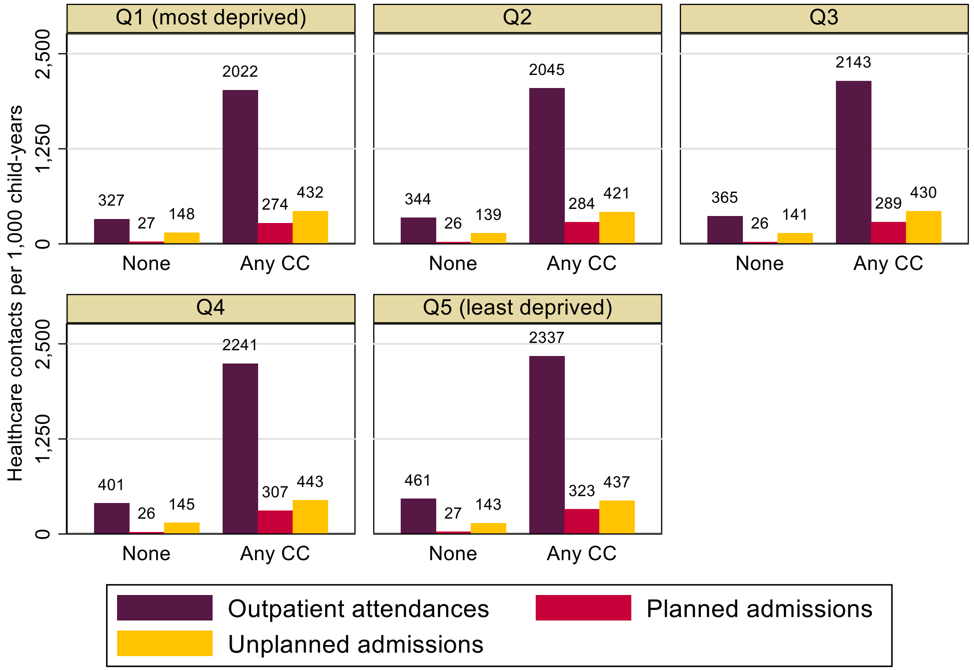

CC – Chronic conditions

Supplementary Figure 5: Reduction in care during the pandemic (March 2020-2021), estimated from predicted minus observed rates of hospital contacts per 1,000 child-years for children aged 0 to 4 years, by ethnic group and presence of a chronic condition.

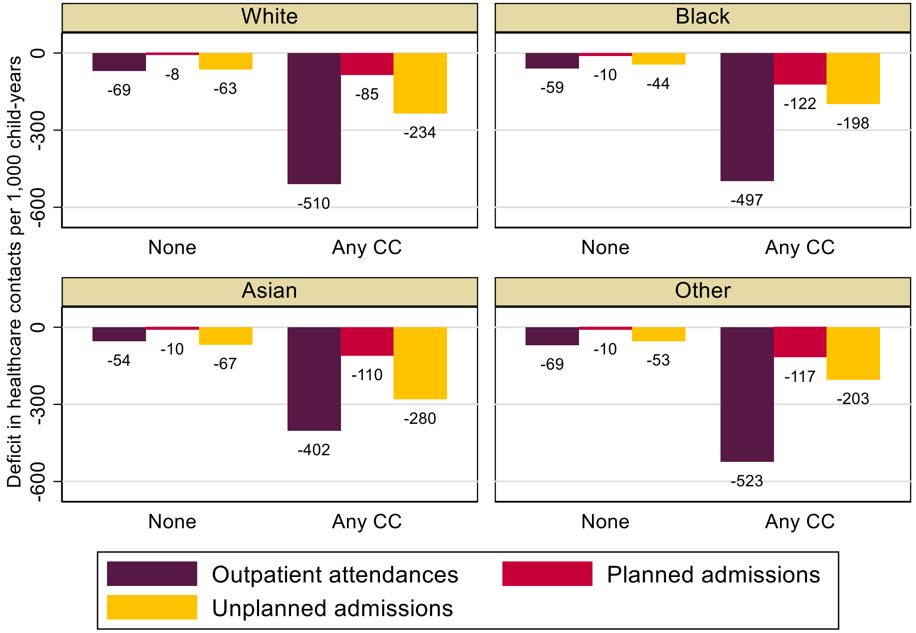

CC – Chronic conditions

Supplementary Figure 6: Reduction in care during the pandemic (March 2020-2021), estimated from predicted minus observed rates of hospital contacts per 1,000 child-years for children aged 0 to 4 years, by deprivation quintile and presence of a chronic condition.

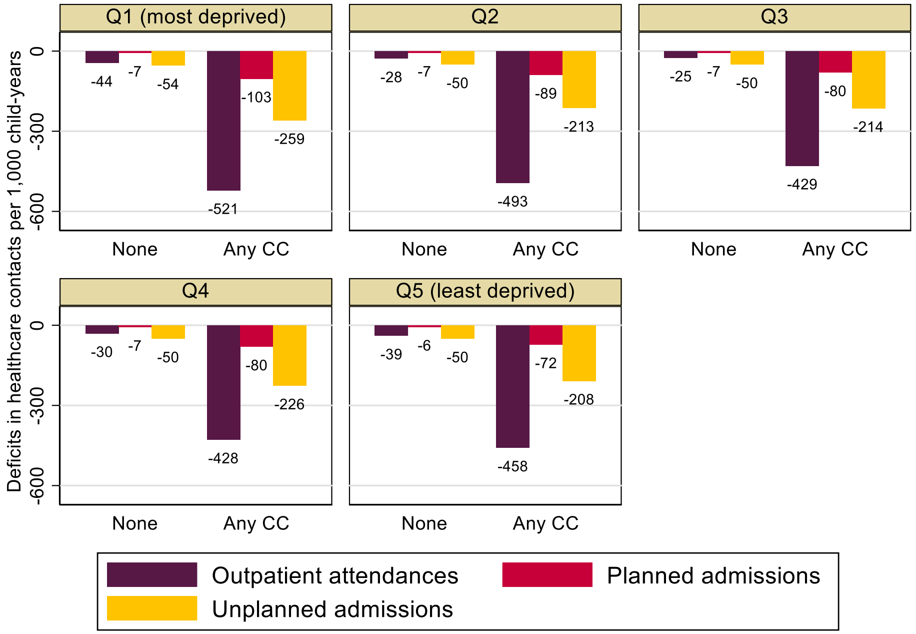

CC – Chronic conditions

Supplementary Figure 7: Weekly difference in observed and predicted hospital contacts among children aged 0 to 4 years during the pandemic (March 2020-2021), by presence of a chronic condition.

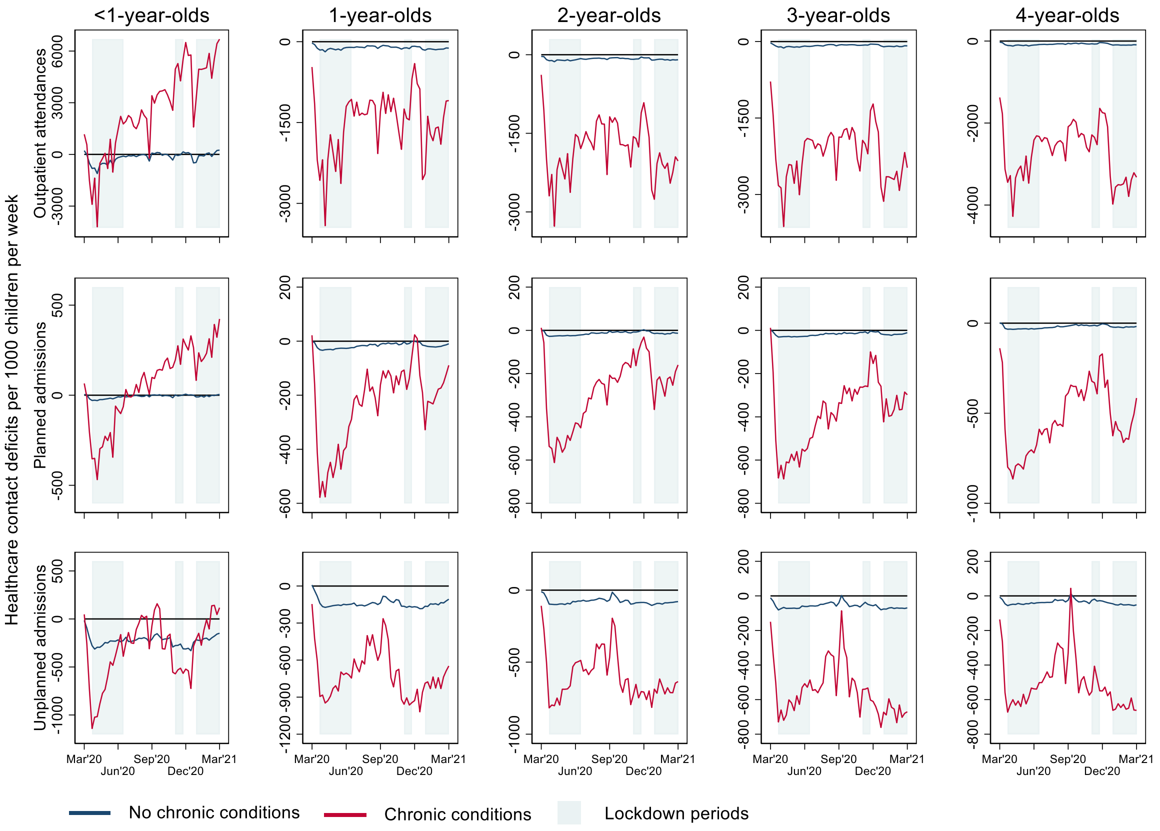

Supplementary Figure 8: Weekly rates of hospital contacts among children aged 0 to 4 years during the pandemic (March 2020-2021) and on average from 2015-2019, by age comparing children in the most and least deprived IMD quintiles.

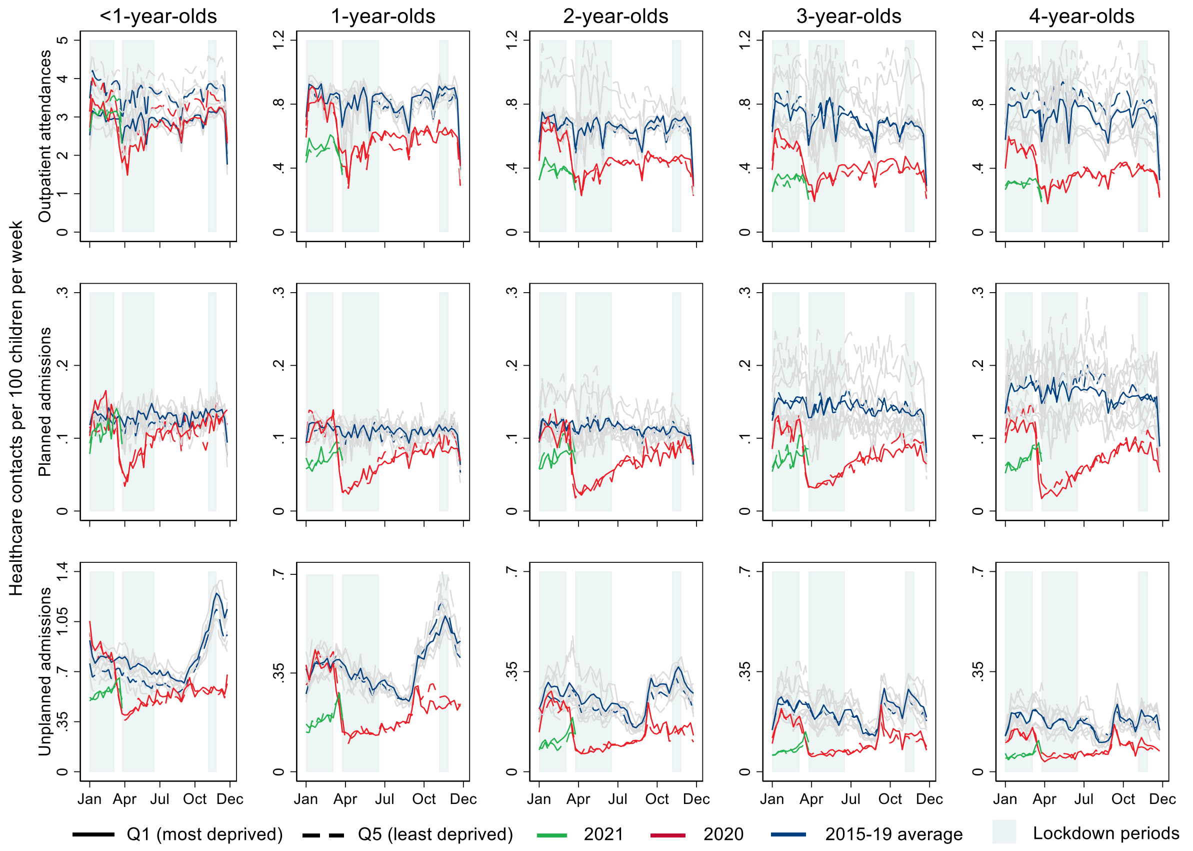

Note: Lockdown 1: March 23 – June 23, 2020; Lockdown 2: November 5 – December 6, 2020; Lockdown 3: January 1 – March 8, 2021.

Supplementary Figure 9: Weekly difference in observed and predicted hospital contacts among children aged 0 to 4 years during the pandemic (March 2020-2021), by deprivation quintile.

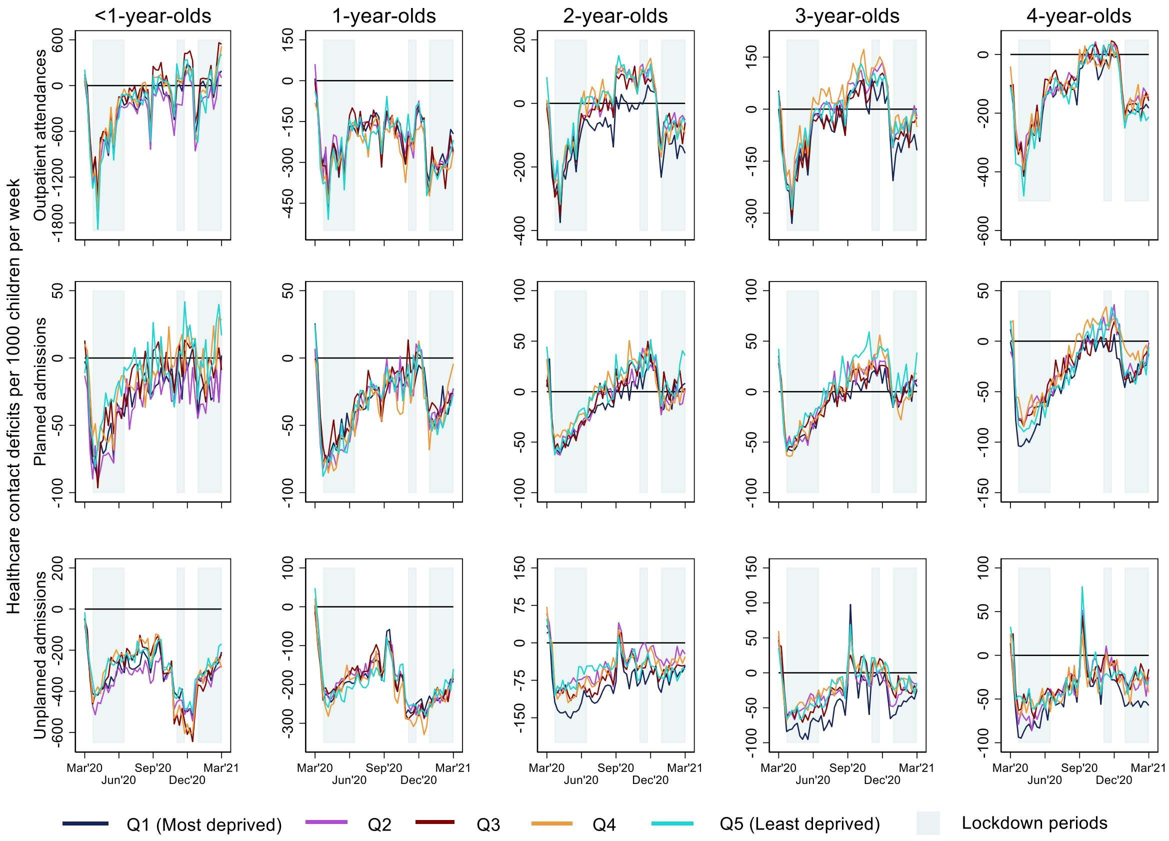

Supplementary Figure 10: Weekly rates of hospital contacts among children aged 0 to 4 years during the pandemic (March 2020-2021) and on average from 2015-2019, by age comparing children of White and Asian ethnic groups.

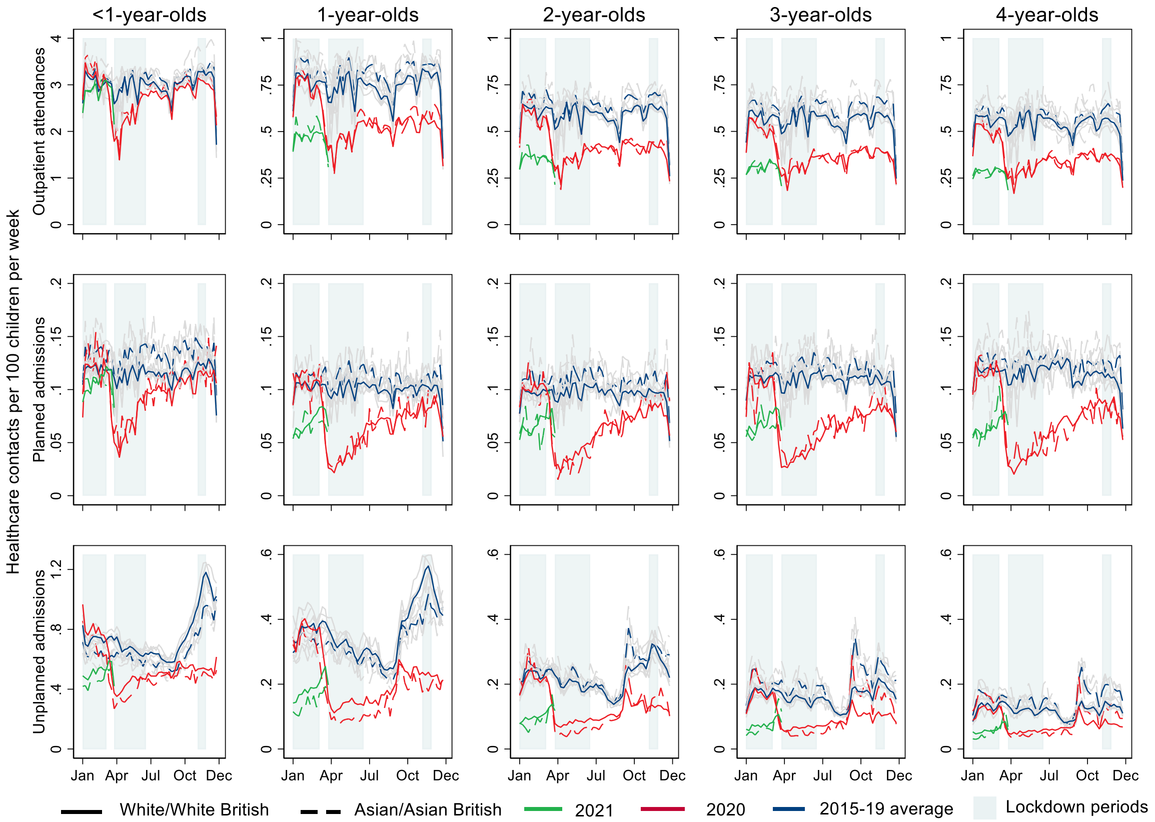

Note: Lockdown 1: March 23 – June 23, 2020; Lockdown 2: November 5 – December 6, 2020; Lockdown 3: January 1 – March 8, 2021.

Supplementary Figure 11: Weekly rates of hospital contacts among children aged 0 to 4 years during the pandemic (March 2020-2021) and on average from 2015-2019, by age comparing children of White and Black ethnic groups.

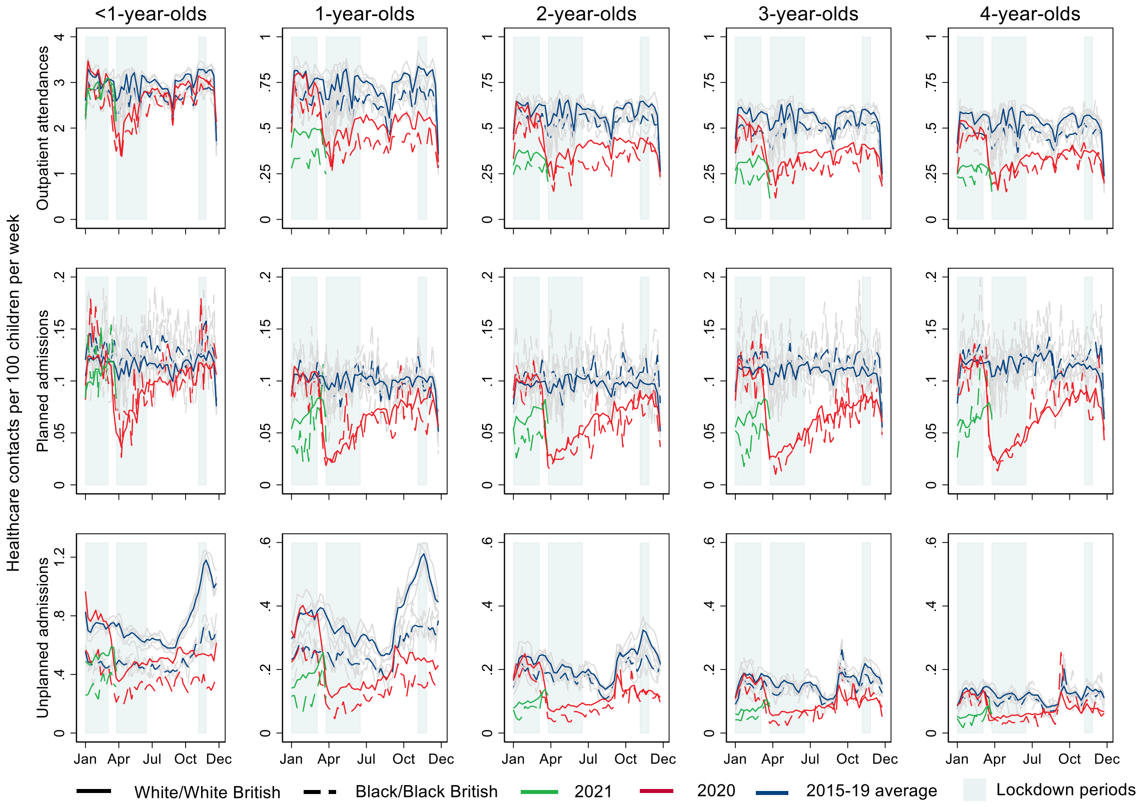

Note: Lockdown 1: March 23 – June 23, 2020; Lockdown 2: November 5 – December 6, 2020; Lockdown 3: January 1 – March 8, 2021.

Supplementary Figure 12: Weekly difference in observed and predicted hospital contacts among children aged 0 to 4 years during the pandemic (March 2020-2021), by ethnic group.

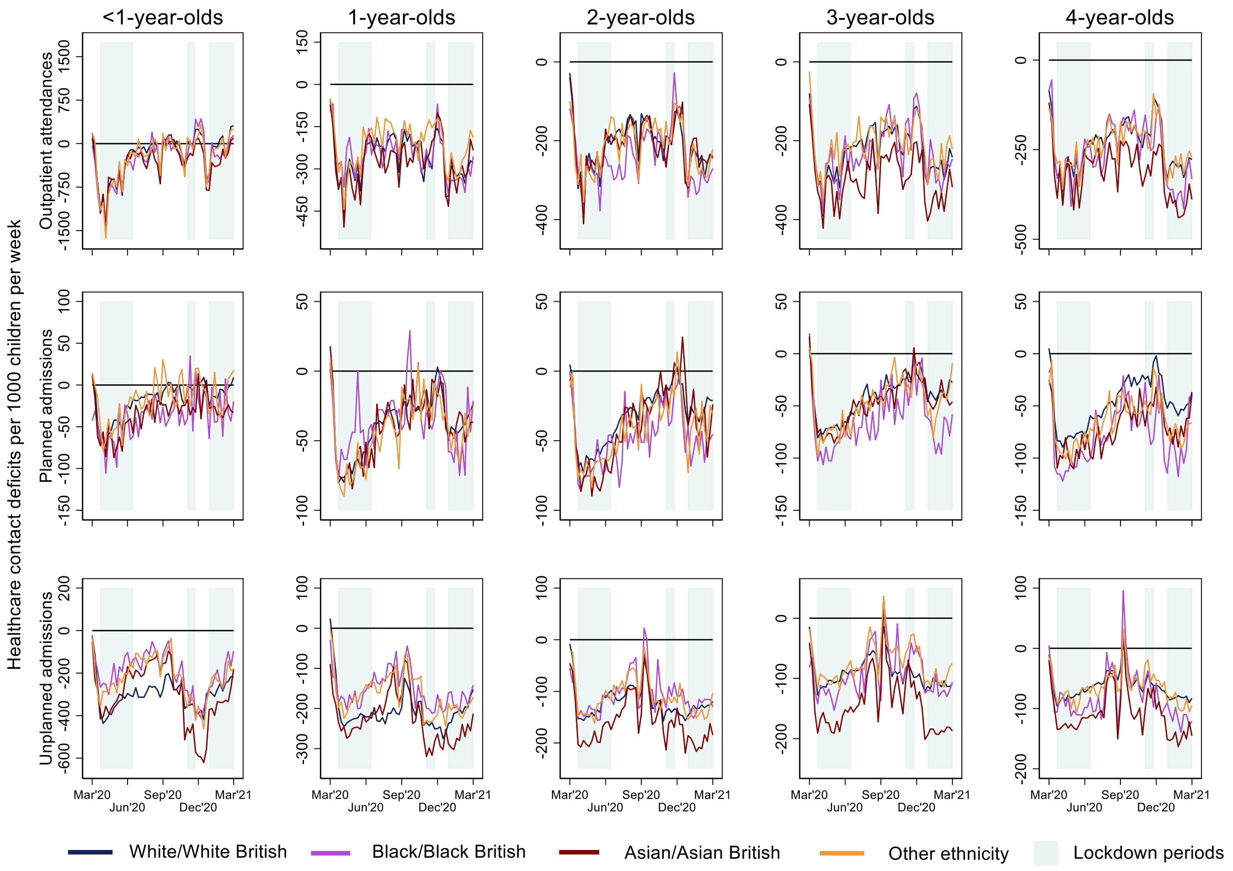

Supplementary Figure 13: Weekly rates of in-person outpatient appointments among children aged 0 to 4 years during the pandemic (March 2020-2021) and on average from 2015-2019, by age.

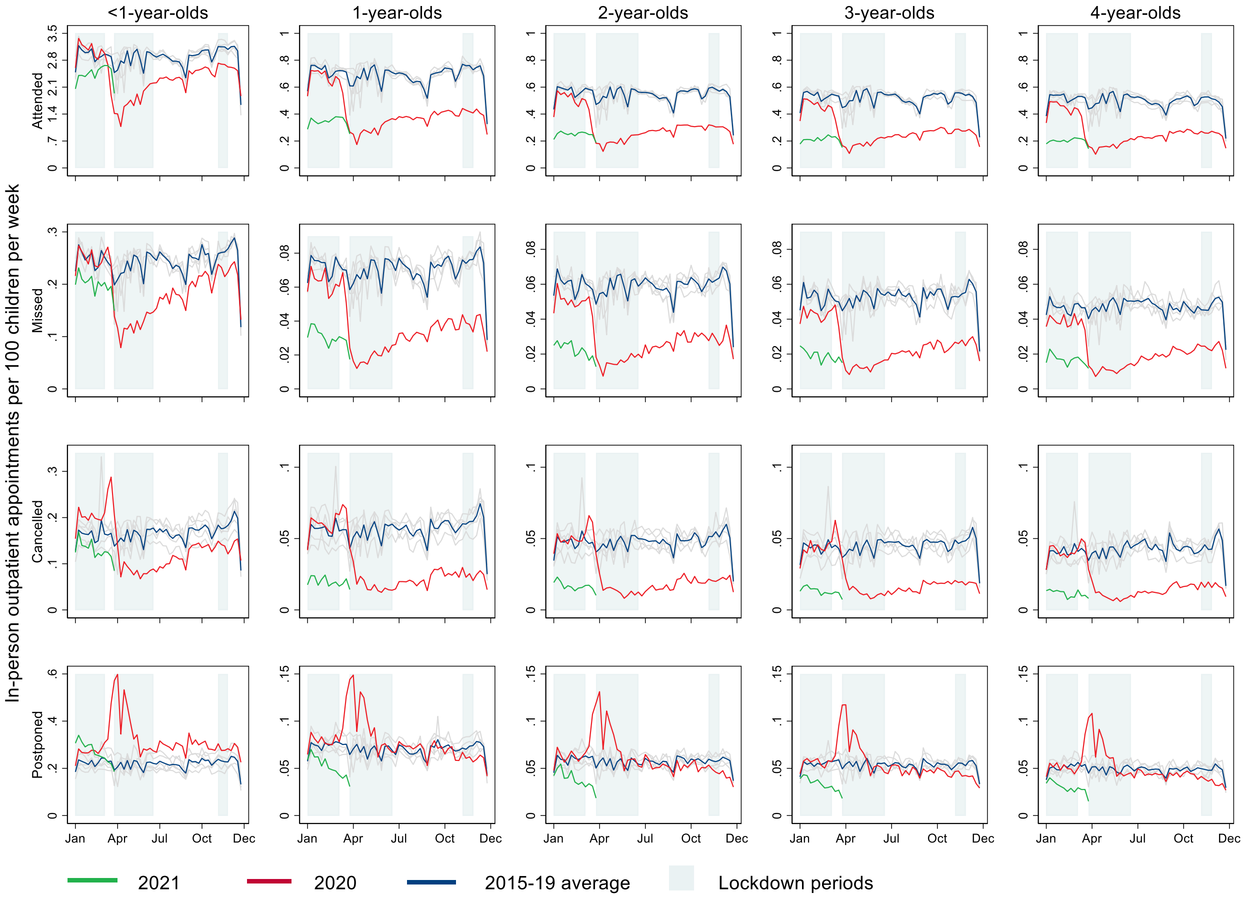

Note: Lockdown 1: March 23 – June 23, 2020; Lockdown 2: November 5 – December 6, 2020; Lockdown 3: January 1 – March 8, 2021.

Supplementary Figure 14: Weekly rates of outpatient attendances (In-person vs tele/virtual) among children aged 0 to 4 years during the pandemic (March 2020-2021) and on average from 2015-2019, by age.

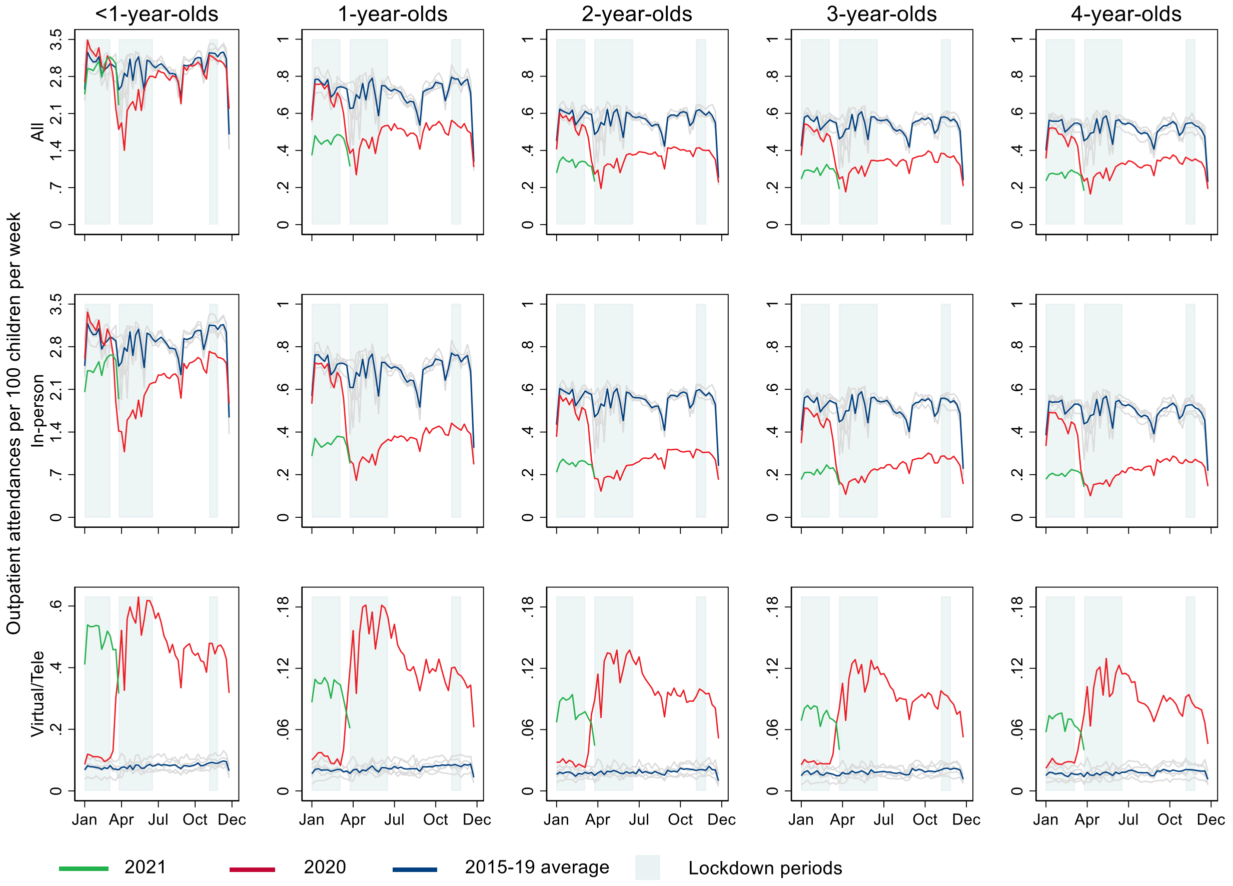

Note: Lockdown 1: March 23 – June 23, 2020; Lockdown 2: November 5 – December 6, 2020; Lockdown 3: January 1 – March 8, 2021.
